# The complete mitochondrial genome of *Numenius minutus* (Charadriiformes: Scolopacidae): comparative and phylogenetic analysis

**DOI:** 10.1080/23802359.2022.2148488

**Published:** 2022-11-23

**Authors:** Peng Chen, Jiaqi Li, Jianliang Zhang, Wei Liu

**Affiliations:** Ministry of Ecology and Environment, Nanjing Institute of Environmental Sciences, Nanjing, China

**Keywords:** Mitochondrial genome, *Numenius minutus*, phylogenetic analysis

## Abstract

In this study, we used the next-generation sequencing method to obtain mitochondrial DNA (*mt*DNA) of *Numenius minutus* Gould 1841 in Scolopacidae, after which we analyzed the structure and phylogeny of the Charadriiformes. The complete *mt*DNA was 17,047 bp in length, and contained 13 protein-coding genes (PCGs), 22 transfer RNA (tRNA) genes, two ribosomal RNA genes, and one control region (CR). The gene structure and arrangement of the mitochondrial genome of 64 Charadriiformes species were basically the same as most birds. The reconstructed phylogenetic tree demonstrated that *Numenius* species were sister groups and monophyletic in Scolopacidae.

Charadriiformes is a complex group, mainly including small- and medium-sized waders, curlew, and gulls (Sibley et al. 1988). *Numenius minutus* Gould 1841 is the smallest *Numenius* and listed as National key protected species in China. There were some reports on mitochondrial genome sequencing and phylogenetic development in Charadriiformes birds (Hu et al. 2017; Guo et al. 2021). Based on 12 PCGs markers, Bayesian’s inference (BI) and maximum-likelihood (ML) trees of 40 species were constructed to classify Charadriiformes into Charadrii, Lari, and Scolopaci (Hu et al. 2017). Up to now, more new species or distributional records in Charadriiformes were found (Wang et al. 2019). In this study, we determined the complete mitochondrial DNA (*mt*DNA) sequence of *N. minutus*. We analyzed the phylogenetic relationship and classification among 64 Charadriiformes species.

In this study, we sequenced the *mt*DNA of *N. minutus* via the next-generation sequencing. After *N. minutus* was captured and banded, blood samples were gathered from Yangkou Village (32.552853322°N, 121.028979706°E), Nantong City, Jiangsu Province, China. These samples (specimen voucher: NIES20191003LQGDHLS01) were deposited into Nanjing Institute of Environment Sciences (https://www.nies.org/). For more information about this voucher, please contact Peng Chen (email: Pengchen12138@163.com). Genomic DNA was prepared in 150 bp paired-end libraries, and analyzed with the high-throughput Illumina NovaSeq6000 platform (Novogene Bioinformatics Technology Co. Ltd., Tianjin, China), yielding about 15 Gb raw reads (Shi et al. 2021; Kongkachana et al. 2022). The mitogenome as a byproduct of whole-genome sequencing was assembled by the NOVO Plasty 3.7 and annotated by MITOS Web Server (Matthias et al. 2013; Dierckxsens et al. 2017). Mitochondrial complete genomes of 63 Charadriiformes species were retrieved from GenBank. We used MEGA 11 and DNASTAR to analyze the mitochondrial genome sequence and base content (Burland 2000; Tamura et al. 2021; Liu et al. 2022).

The complete mitochondrial genome of *N. minutus* (GenBank accession number: OK552672) was a circular molecular with 17,047 bp in total length, containing 13 protein-coding genes (PCGs), two ribosomal RNA genes, 22 transfer RNA (tRNA) genes, and one control region (CR). All PCGs in the *mt*DNA use ATG start codons except for COI (GTG) and ND3 (ATA). Six of these PCGs used complete (TAA), two used complete (AGG), one used complete (TAG), one used complete (AGA), and three used incomplete stop codons (T–/T–), which could form TAA by post-transcription polyadenylation (Anderson et al. 1981). The overall nucleotide composition was 25.36% of T, 29.69% of C, 30.31% of A, and 13.64% of G in *N. minutus.* The positive AT skew indicated the nucleotide composition of the complete mitochondrial genome is slightly biased toward A and C.

ML and BI trees were constructed by using the best-fit model of GTR + G+I selected by MrModelTest 3.06 software and PAUP 4.0 software with Akaike information criterion (AIC) (Figure 1) (Nylander et al. 2004). *Gallus gallus* and *Zapornia fusca* were considered as outgroup in the analysis. The results of phylogenetic analysis of Charadriiformes show that the order consists of three branches, which is similar to the results of previous studies. The results of phylogenetic analysis of Charadriiformes showed that there were three main branches, Lari (Sternidae, Laridae, Alcidae, and Stercorariidae) and its sister Scolopaci (Scolopacidae and Jacanidae), which were in turn sister to the suborder Charadrii (Recurvirostridae, Haematopodidae, and Charadriidae), which were similar to the results of previous studies (Smith and Clarke 2015; Cheng 2017; Hu et al. 2017). In the Lari, our phylogenies indicated that Laridae and Sternidae were clustered in a sister group. In the Scolopaci, there are many species and many branches, *N. minutus* and *N. phaeopus* were clustered in a sister group, and *N. madagascariensis* and *N. tenuirostris* were sister group. In the Charadrii, Recurvirostridae and Haematopodidae were sister group, and were in turn sister to Charadriidae. Based on more information, the evolutionary analysis among Charadriiformes species is more comprehensive, and unravels the phylogenetic relationship between *N. minutus* and other species (Jetz et al. 2012; Hu et al. 2017). Moreover, high posterior probability value indicates a high degree of credibility. In future, we look forward to obtaining more mitochondrial genome so that to enhance the knowledge of evolutionary history in Charadriiformes.

**Figure 1.**
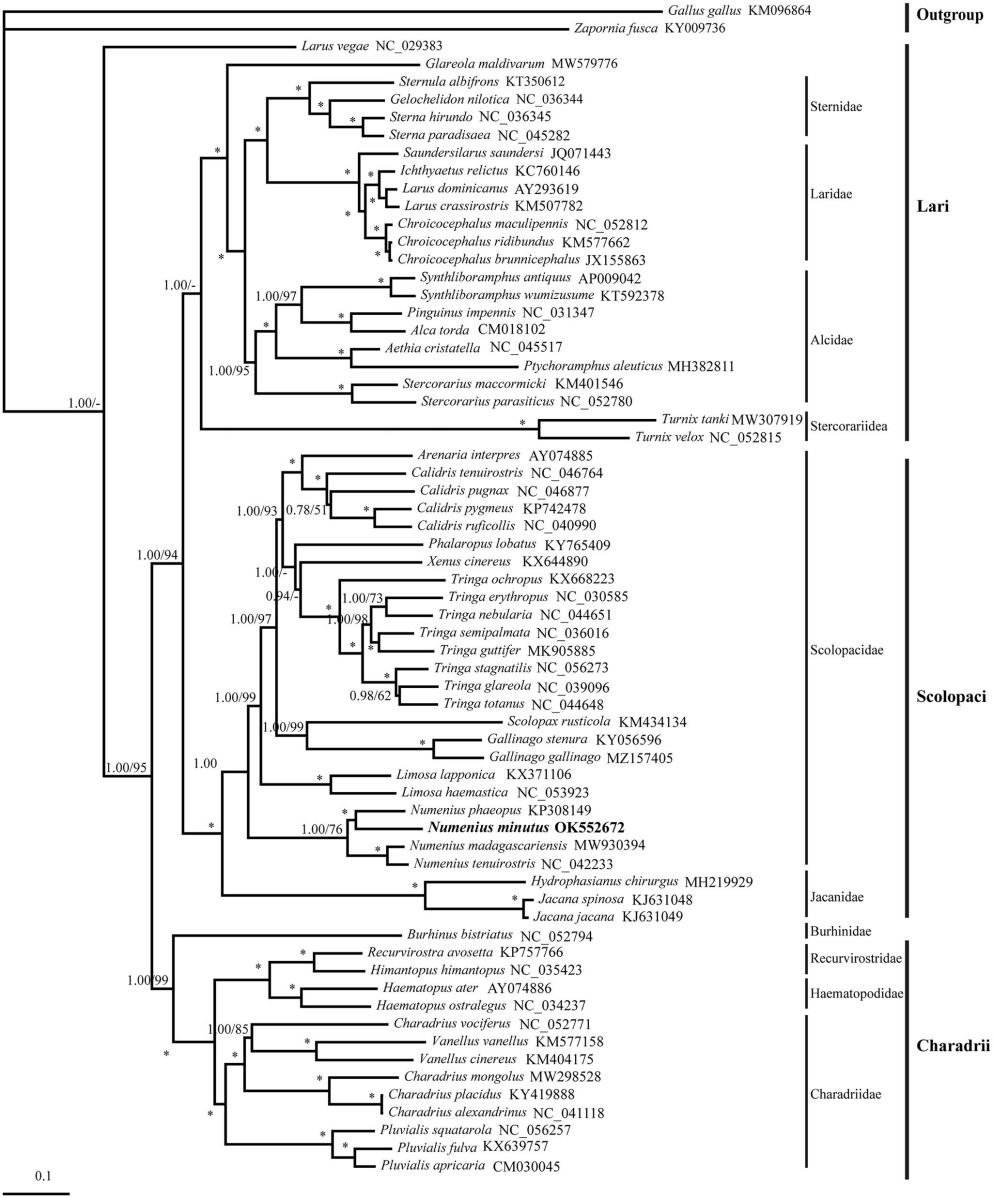
BI and ML trees constructed based on complete *mt*DNA sequences of Charadriiformes. Numbers represent bootstrap values (BI/ML) and only those >60% are shown. Asterisks indicate posterior probabilities of 100%. *Numenius minutus* in this study is bold.

## Data Availability

The mitogenome data supporting this study have been deposited in GenBank under the accession number OK552672 and is also openly available at https://www.ncbi.nlm.nih.gov/nuccore/OK552672.1. The associated BioProject, SRA, and Bio-Sample numbers are PRJNA766551, SRR16081381, and SAMN21851017, respectively.
